# Real-Time Acute Kidney Injury Risk Stratification–Biomarker Directed Fluid Management Improves Outcomes in Critically Ill Children and Young Adults

**DOI:** 10.1016/j.ekir.2023.09.019

**Published:** 2023-09-22

**Authors:** Stuart L. Goldstein, Kelli A. Krallman, Jean-Philippe Roy, Michaela Collins, Ranjit S. Chima, Rajit K. Basu, Lakhmir Chawla, Lin Fei

**Affiliations:** 1 Cincinnati Children’s Hospital Medical Center, Cincinnati, Ohio, USA; 2 Lurie Children’s Hospital, Chicago Illinois, USA; 3 Department of Veteran’s Affairs, Washington, DC, USA

**Keywords:** acute kidney injury, children, continuous renal replacement therapy, neutrophil gelatinase associated lipocalin, renal angina index

## Abstract

**Introduction:**

Critically ill admitted patients are at high risk of acute kidney injury (AKI). The renal angina index (RAI) and urinary biomarker neutrophil gelatinase-associated lipocalin (uNGAL) can aid in AKI risk assessment. We implemented the Trial in AKI using NGAL and Fluid Overload to optimize CRRT Use (TAKING FOCUS 2; TF2) to personalize fluid management and continuous renal replacement therapy (CRRT) initiation based on AKI risk and patient fluid accumulation. We compared outcomes pre-TF2 and post-TF2 initiation.

**Methods:**

Patients admitted from July 2017 were followed-up prospectively with the following: (i) an automated RAI result at 12 hours of admission, (ii) a conditional uNGAL order for RAI ≥8, and (iii) a CRRT initiation goal at 10% to 15% weight-based fluid accumulation.

**Results:**

A total of 286 patients comprised 304 intensive care unit (ICU) RAI+ admissions; 178 patients received CRRT over the observation period (2014–2021). Median time from ICU admission to CRRT initiation was 2 days shorter (*P* < 0.002), and ≥15% pre-CRRT fluid accumulation rate was lower in the TF2 era (*P* < 0.02). TF2 ICU length of stay (LOS) after CRRT discontinuation and total ICU LOS were 6 and 11 days shorter for CRRT survivors (both *P* < 0.02). Survival rates to ICU discharge after CRRT discontinuation were higher in the TF2 era (*P* = 0.001). These associations persisted in each TF2 year; we estimate a conservative $12,500 health care cost savings per CRRT patient treated after TF2 implementation.

**Conclusion:**

We suggest that automated clinical decision support (CDS) combining risk stratification and AKI biomarker assessment can produce durable reductions in pediatric CRRT patient morbidity.

## Graphical abstract

**Figure:**
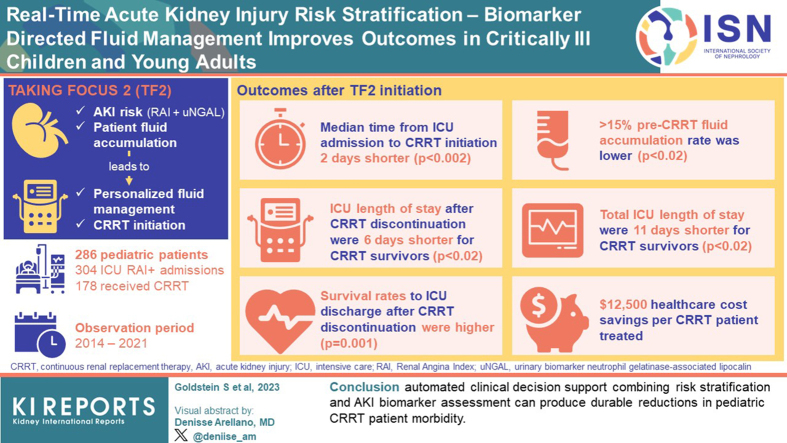

AKI occurs in 10% to 40% of children and young adults admitted to a pediatric ICU. Severe AKI (stage 2 or 3 defined by Kidney Disease Improving Global Outcomes criteria)^1^ is associated with increased risk of morbidity and mortality.^2^, ^3^, ^4^, ^5^ We spent the past 12 years validating an AKI risk stratification system, the RAI.^6^ The RAI, calculated at 12 hours after ICU admission, performs well to predict severe AKI presence at 72 hours.^6^, ^7^, ^8^ Meta-analysis of 11 studies comprising 3701 children demonstrated RAI predictive performance (AUC-ROC) of 0.88 (95% confidence interval 0.85–0.91), sensitivity 0.85 (95% confidence interval 0.74–0.92) and specificity 0.79 (95% confidence interval 0.69–0.89).^9^

Integration of uNGAL concentrations with RAI improved AKI prediction in a pilot study at our center.^7^ We recently reported first-year postimplementation results of our automated RAI-uNGAL CDS program to predict severe AKI at 72 hours of ICU admission.^10^ uNGAL integration improved RAI positive predictive value and specificity without decrease in negative predictive value. Meta-analysis of 4 RAI-uNGAL studies comprising 1523 patients validated AKI prediction enrichment with RAI-uNGAL integration.^11^

Although improved AKI prediction is essential, prognostic enrichment must be coupled with clinical decision making to refine diagnostics, assess therapeutics, and/or direct personalized care to achieve health care benefits.^12^ Current AKI management, including provision of CRRT, remains supportive; however, patients who require CRRT remain at 5-fold increased mortality risk.^4^ Multiple well-designed randomized prospective studies in critically ill adults have assessed if provision of “earlier versus later (or accelerated vs. standard)” CRRT initiation leads to improved patient outcomes.^13^, ^14^, ^15^, ^16^ These studies randomized patients, for the most part, based on Kidney Disease Improving Global Outcomes Stage and time to CRRT initiation after a patient met the inclusion criteria, with the largest 3 demonstrating no improvement in patient survival, and worse outcomes in some patients allocated to early initiation.

The pediatric critical care nephrology community, in contrast, has considered CRRT initiation “timing” based on degree of fluid accumulation at CRRT initiation. Indeed, the pediatric literature is replete with 2 decades of observational studies, aggregated into a recent systematic review, showing consistent associations between fluid accumulation thresholds at CRRT initiation and poor outcomes.^17^ Interestingly, a recent single center *post hoc* study demonstrated that an RAI ≥ 8 was associated with increased risk of developing ≥15% fluid accumulation by day 3 of ICU admission (adjusted odds ratio 5.1, 95% confidence interval 1.23–21.2, *P* = 0.025).^18^ A reasonable critique of this literature, including from the Pediatric Acute Disease Quality Initiative consensus conference,^12^ is that these associations do not equate to causation, and therefore, a fluid accumulation prevention strategy needs to be tested prospectively to validate assumptions regarding benefits of CRRT initiation timing based on fluid accumulation status.

We implemented the TF2 in 2017, to personalize fluid management, and standardize diuretic response assessment and CRRT initiation based on AKI risk and patient fluid accumulation. We compared CRRT cohorts before and after TF2 implementation to assess differences in TF2 patient-centered outcomes and health care costs.

## Methods

TF2 methods have been reported elsewhere.^19^ All patients admitted to the Cincinnati Children’s Hospital Medical Center pediatric ICU are entered into the pathway. Exclusions included the following: admission for <48 hours, chronic kidney disease stage IV/V, an active do not resuscitate order, or RRT prior to ICU admission or patients age >25 years (*n* = 12, 2.6%). TF2 was approved by our institutional review board as an observational study, receiving a waiver for informed consent and registered on clinicaltrials.gov (NCT03541785) prior to patient enrollment.

### Study Flow

The Pre-TF2 AKI and CRRT decision process and TF2 RAI-uNGAL CDS flow are depicted in Supplementary Figure S1. RAI is calculated automatically at 12 hours after ICU admission and results are immediately populated in the electronic health record. RAI is the product of demographic risk (ICU admission = 1, stem cell/solid organ transplant recipient = 3, invasive mechanical ventilation and 1 intravenous vasoactive medication = 5) and degree of physiological change (increase in serum creatinine [SCr] or positive fluid accumulation [1, 2, 4, or 8]). The algorithm identifies the lowest SCr value within 90 days before admission for baseline. If none is found, it imputes baseline SCr based on the most recent patient height within the previous year, assuming an estimated glomerular filtration rate of 120 ml/min per 1.73 m^2^, as validated in the pediatric literature^4^^,^^20^:

Imputed SCr (mg/dl) = 0.413 × patient height (cm)/120 ml/min per 1.73 m^2^,^21^

If height is unavailable, age is used to impute baseline SCr with similar accuracy, as validated earlier in this project.^22^ For RAI < 8, the patient receives the clinical standard of care, with no action directed by TF2. However, clinicians could order a uNGAL test based on their individual clinical judgement (i.e., TF2 did not prohibit uNGAL testing for patients with an RAI < 8).

All ICU admission order sets have a conditional order for uNGAL (The NGAL Test assay (BioPorto Diagnostics, Denmark) that releases automatically for RAI ≥ 8 (range [1–40]). Values are reported between <50 and 15,000 ng/ml by manual dilution. The uNGAL result is populated automatically within 2 hours in the electronic health record. In addition, clinicians could order serial uNGAL tests based on their individual clinical judgement (i.e., TF2 did not prohibit additional uNGAL testing for patients with an RAI ≥ 8 after the initial uNGAL result).

After the uNGAL result is posted, fluid, diuretic management, and initiation of CRRT are guided by the CDS algorithm. For results <150 ng/ml, the patient receives the clinical standard of care, with no action directed by TF2. For results ≥500 ng/ml, the ICU team considers fluid restriction, and a furosemide stress test (1 mg/kg bolus infusion of furosemide; 1.5 mg/kg if a diuretic was given in the previous 24 hours), and/or consideration for CRRT initiation as needed to prevent fluid accumulation of >15% of body weight at ICU admission:

Fluid accumulation from ICU admission to CRRT initiation^23^ = $\frac{\text{Fluid}\;\text{in}\;\left(l\right)-\text{Fluid}\;\text{out}\;\left(l\right)}{\text{ICU}\;\text{admission}\;\text{weight}\;\left(\text{kg}\right)}\times 100\%$

We chose a 15% fluid accumulation threshold because the largest pediatric study assessing fluid accumulation revealed 8-fold increased mortality risk for CRRT initiation at >20% fluid accumulation.^24^ For uNGAL results between 150 and 500 ng/ml, the patient receives a furosemide stress test. Based on urine output response, the patient continues receiving diuretics or consideration for CRRT initiation to prevent fluid accumulation of >15%. We chose these uNGAL thresholds because of the following reasons: (i) 50 ng/ml is the lower limit of assay detection, (ii) 150 ng/ml is associated with AKI prediction in numerous pediatric populations, and (iii) 500 ng/ml is associated with a high specificity for severe AKI.^25^, ^26^, ^27^, ^28^ The ultimate decision to initiate CRRT was made by the treating clinical team and not mandated by the TF2 pathway.

### Statistical Analysis

Data reported represent the first 3 stages of the TF2 CDS pathway as follows: (i) automated RAI calculation, (ii) RAI+ directed automatic NGAL assessment, and (iii) fluid accumulation-based CRRT initiation. We do not report furosemide stress test data here.

We based our target sample numbers on historical annual rates of 700 Cincinnati Children’s Hospital Medical Center ICU patient admissions lasting more than 48 hours. Data from our single center and multicenter studies demonstrate that 10% to 13% of these admissions will be complicated by severe AKI (Kidney Disease Improving Global Outcomes stage 2 or 3), which has been independently associated with morbidity and mortality. Multicenter data show that 24% of all patients admitted to a pediatric ICU will develop 10% fluid overload in 7 days. We aimed to screen 2100 patients over 3 years with the resultant expectation that 210 will develop severe AKI, 150 of these will have a uNGAL >150 ng/ml, 100 of whom will develop >10% fluid accumulation and 50 of whom will require CRRT.

The population is comprised of patients who received CRRT for 3.5 years pre-TF2 (January 2014–June 2017) versus 4 years post-TF2 implementation (July 2017–June 2021). We divided outcome assessments according to 3 temporal phases of the CRRT course for each patient:

1. ICU admission to CRRT initiation: time (days), fluid accumulation (% of ICU admission weight), rate of ≥ 15% fluid accumulation
2. CRRT course: duration (days), survival to CRRT discontinuation (%)
3. ICU discharge outcomes: CRRT discontinuation to ICU discharge duration (days), ICU LOS (days), survival to ICU discharge (%)

The ICU discharge date was defined as the date the patient was transferred out of the ICU, and not the date and time the order for transfer was logged in the electronic health record. Given that patient death during CRRT represents a competing outcome for CRRT duration, CRRT discontinuation to ICU duration, and ICU LOS, potentially biasing outcomes for early versus late patient death in one era versus another, we only compared these pre-TF2 and post-TF2 implementation outcomes for CRRT survivors.

We performed subset analyses for the following: (i) patients initiating CRRT within 14 days of ICU admission, because 82% of children receive CRRT in this timeframe,^29^ and outcome associations with TF2 implementation past 14 days cannot be justified, and (ii) the first ICU admission requiring CRRT to limit potential bias for one era from survival leading to repeat admissions in that era. We compared RAI+ rates between pre-TF2 (calculated manually) and TF2 eras (automated by CDS) to assess differences in AKI risk. Patient illness severity at ICU admission was defined by Pediatric Risk of Mortality III score.^30^

We compared 2 different time periods over a 7.5-year span; therefore, outcomes could be confounded by temporal changes in the following: (i) case-mix, (ii) ICU policies (admission to ICU/withholding strategies), and/or (iii) general center performance independent of the protocol. To address such era confounding effects, we compared baseline characteristics carefully including RAI scores, and performed year-by-year outcome analysis.

We assessed health care cost differences between eras using fiscal year 2023 costs for ICU and CRRT care. For the most conservative estimate, we used data for patients initiating CRRT within 14 days of ICU admission. ICU costs ($2448 per day) included registered nurse and respiratory therapist salary plus fringe cost, and supply costs for ICU support with mechanical ventilation; CRRT costs (average daily cost $194.88) included disposable costs (CRRT filter circuits, fluids, supplies, and cost for the NGAL test) and CRRT registered nurse hourly salary plus fringe benefits for setting up or monitoring CRRT. Physician costs were not included. Individual medication and disposable costs are included in the average daily ICU cost.

We assessed uNGAL integration with the RAI+ in the TF2 era to improve predictive performance for day 2 to 4 severe AKI (including a subset analysis for biological sex based on external anatomy given reported higher uNGAL ranges in females).^31^ The uNGAL concentration was presumed to be >500 ng/ml for anuric patients 12 hours after admission. Descriptive statistics are summarized for categorical and continuous variables. Fisher’s exact test was used to assess significance of binomial outcomes. Statistical analyses were performed using SAS Software 9.4. (SAS Institute Inc., Cary, NC) and R-package MatchIt 4.5.3 (R Core Team 2021, Vienna, Austria).

## Results

**Figure 1:**
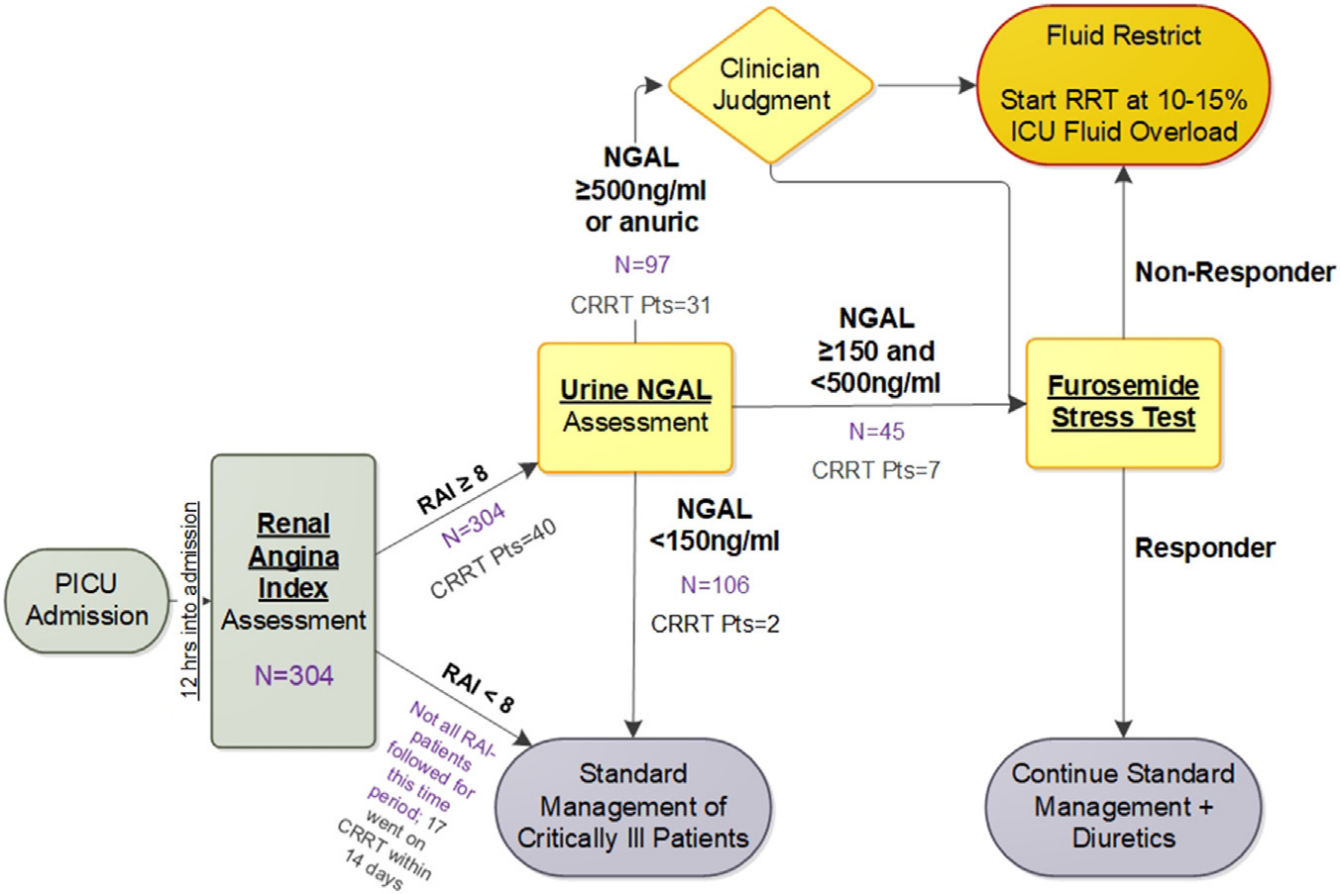
The TAKING FOCUS 2 Clinical Decision Support Pathway. The current manuscript incorporates all components of the pathway except for the furosemide stress test. ICU, intensive care unit; NGAL, neutrophil gelatinase-associated lipocalin; PICU, pediatric intensive care unit; RAI, renal angina index; RRT, renal replacement therapy.

**Figure 2:**
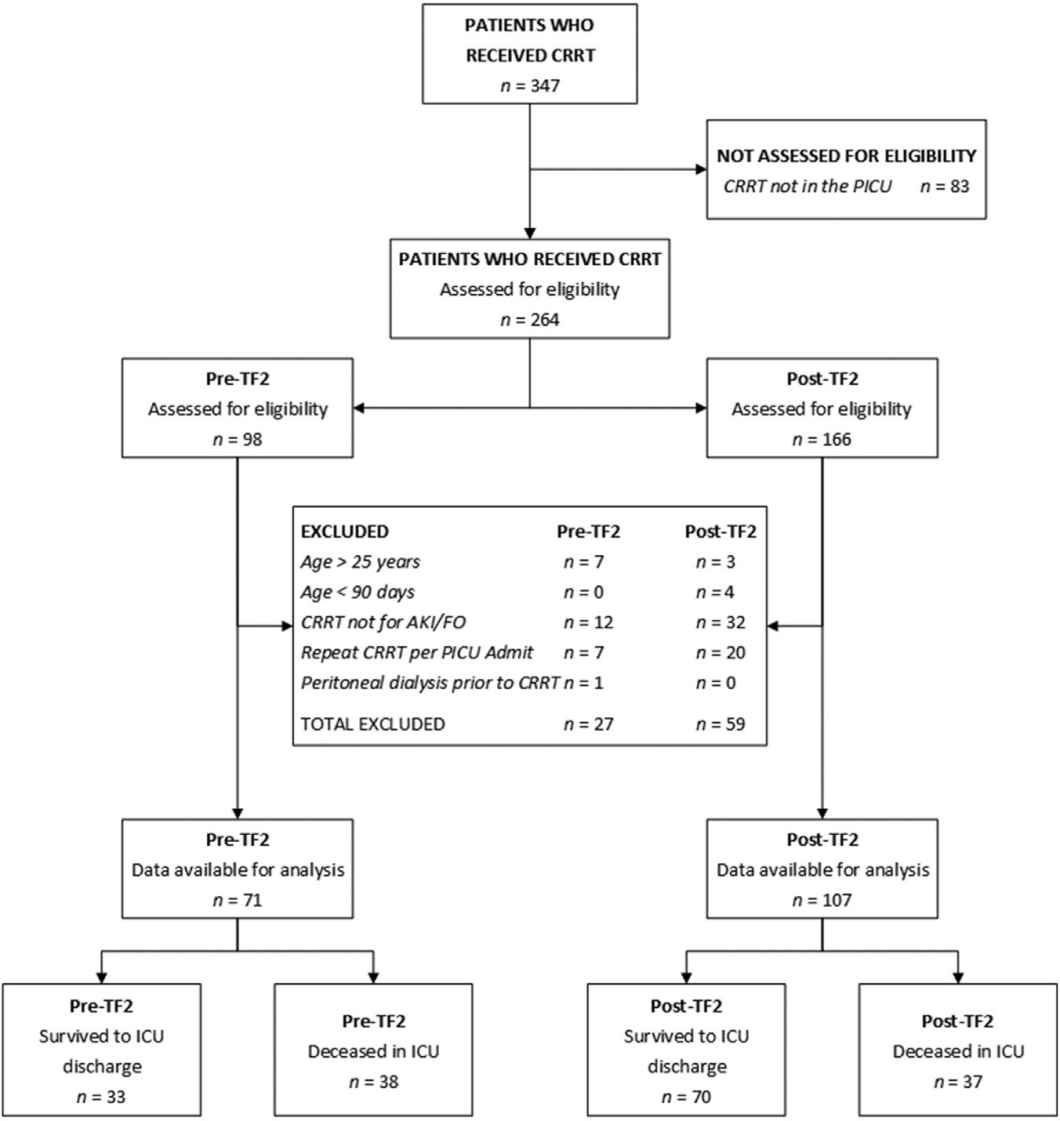
CONSORT Diagram for patients on CRRT according to inclusion and exclusion criteria by TF2 era. CRRT, continuous renal replacement therapy; PICU, pediatric intensive care unit; TF2, TAKING FOCUS 2.

Patient distribution along TF2 is depicted in Figure 1. A total of 286 patients comprised 304 ICU RAI+ admissions (RAI ≥ 8); 247 patients were 3 months to 18 years of age, 39 were 18 to 25 years of age. Of the 304 RAI+ admissions, 102 (33.6%) required imputation to estimate a baseline SCr concentration. Mean annual ICU admissions were similar (2620 vs. 2515) in the pre-TF2 versus TF2 eras. Patient demographic data for unique RAI+ patients are presented in Table 1. The CRRT patient cohort distribution showing rationale for inclusion and exclusion is depicted in Figure 2.

**Table 1:** Subject demographics and outcomes of TF2 at first PICU admission

Demographics	Unique patients (*N* = 286)
Sex	
Female	128 (44.8%)
Male	158 (55.2%)
Age (yrs)	
Median [IQR]	10.9 [2.9, 16]
(Min, Max)	0.26, 24.4
Race	
Asian/Native American	3 (1.0%)
Black	53 (18.5%)
Mixed	24 (8.4%)
White	206 (72%)
Hispanic	18 (6.4%)
BMT	46 (16.1%)
Solid organ transplant	62 (21.7%)
Survival to PICU D/C	253 (88.5%)
Day 28 survival	254 (88.8%)
Survival to hospital D/C	224 (78.3%)

BMT, bone marrow transplant; IQR, interquartile range; PICU, pediatric intensive care unit; TF2, TAKING FOCUS 2.

### ICU Admission to CRRT Initiation

Data from the 178 patients who received CRRT over the 7.5-year observation period were available for analysis: 71 pre-TF2 (median 25 patients/yr) period and 107 post-TF2 (median 23 patients/yr) implementation. Patient demographic, pre-CRRT clinical, and outcome data are compared in Table 2. Patient age, weight, or Pediatric Risk of Mortality III score did not differ between the 2 eras. Median time from ICU admission to CRRT initiation was shorter (2.2 days, *P* = 0.002); ICU to CRRT fluid accumulation (%) and ≥15% fluid accumulation rates were lower in the TF2 era (*P* = 0.0008 and *P* = 0.02, respectively) for the total cohort. All associations were maintained in subset analyses of patients receiving CRRT in the first 14 days of ICU admission (Table 2), for their first RAI+ admission (Supplementary Table S1).

**Table 2:** Compairsons between the pre-TF2 and TF2 cohorts

All patients who received CRRT (*N* = 178)				
Variable		Pre-TF2 (*n* = 71)	Post-TF2 (*n* = 107)	*P*-value
Pre-CRRT patient demographics and fluid status				
Patient age (yrs)	Median [IQR]	8.1 [2.0, 15.1]	10.3 [2.3, 17.0]	0.37
Patient PICU admission weight (kg)	Median [IQR]	26.5 [13.3, 49.0]	30.7 [13.2, 59.0]	0.21
PRISM III score at PICU admission	Median [IQR]	12 [8.0, 16]	10 [5.0, 17]	0.23
Renal angina index at 12 hours of PICU admission^a^	Median [IQR]	8 [6, 24]	15 [6, 40]	0.26
Time from PICU admission to CRRT initiation (d)	Median [IQR]	4.5 [2.5, 12.5]	2.3 [1.4, 5.8]	0.002
Fluid accumulation from PICU admission to CRRT initiation (%)	Median [IQR]	12.1 [4.9, 20.3]	4.1 [0.6, 12.2]	0.0008
PICU fluid accumulation >15% at CRRT initiation	Yes	26 (36.6%)	22 (20.6%)	0.02
PICU fluid accumulation >20% at CRRT initiation	Yes	18 (25.4%)	20 (18.7%)	0.35
Patient outcome measures		Pre-TF2 (*n* = 71)	Post-TF2 (*n* = 107)	
Survival to CRRT D/C	Yes	48 (67.6%)	74 (69.2%)	0.87
Survival to PICU D/C	Yes	33 (46.5%)	70 (65.4%)	0.02
		Pre-TF2 (*n* = 48)	Post-TF2 (*n* = 74)	
CRRT duration among CRRT survivors (d)	Median [IQR]	5.8 [2.9, 12.2]	4.0 [1.9, 9.7]	0.06
		Pre-TF2 (*n* = 33)	Post-TF2 (*n* = 70)	
PICU LOS after CRRT D/C among PICU survivors (d)	Median [IQR]	8.6 [4.5, 13]	2.6 [0.7, 8.6]	0.002
Total PICU length of stay among PICU survivors (d)	Median [IQR]	24 [12, 39]	13 [6, 26]	0.02
Patients who received CRRT within 14 days of PICU admission (*N* = 151)				

Variable		Pre-TF2 (*n* = 56)	Post-TF2 (*n* = 95)	*P*-value
Pre-CRRT patient demographics and fluid status				
PRISM III score at PICU admission	Median [IQR]	12 [8.5, 16]	10 [6, 17]	0.28
Time from PICU admission to CRRT initiation (d)	Median [IQR]	2.7 [1.8, 6.1]	1.7 [1.1, 3.9]	0.006
Fluid accumulation from PICU admission to CRRT initiation (%)	Median [IQR]	11.7 [4.8, 20.4]	3.7 [0.5, 12.1]	0.0004
PICU fluid accumulation >15% at CRRT Initiation	Yes	22 (40.0%)	18 (18.9%)	0.007
Patient outcome measures		Pre-TF2 (*n* = 56)	Post-TF2 (*n* = 95)	
Survival to CRRT D/C	Yes	36 (64.3%)	68 (71.6%)	0.37
Survival to PICU D/C	Yes	26 (46.4%)	65 (68.4%)	0.01
		Pre-TF2 (*n* = 36)	Post-TF2 (*n* = 68)	
CRRT duration among CRRT survivors (d)	Median [IQR]	5.8 [3.2, 12.2]	4.2 [2.2, 9.7]	0.07
		Pre-TF2 (*n* = 26)	Post-TF2 (*n* = 65)	
PICU LOS after CRRT D/C among PICU survivors (d)	Median [IQR]	7.9 [2.5, 11]	2.5 [0.6, 6.5]	0.01
Total PICU length of stay among PICU survivors (d)	Median [IQR]	17 [9, 31]	11 [6, 20]	0.08

CRRT, continuous renal replacement therapy; IQR, interquartile range; PICU, pediatric intensive care unit; PRISM, pediatric risk of mortality; TF2, TAKING FOCUS 2.

a *n* = 61 for pre-TF2 and 86 for TF2 eras as patients <3 months of age or with ESRD do not receive an RAI calculation.

Forty-eight pre-TF2 and 74 TF2 patients survived to the time of CRRT discontinuation (67.6% vs. 69.2%, *P* = 0.87). Patient age, weight, Pediatric Risk of Mortality III score, or percent fluid accumulation did not differ between CRRT survivors versus nonsurvivors (Supplementary Table S2).

### CRRT Duration (CRRT Survivors Only)

Median CRRT duration did not differ between the pre-TF2 and TF2 eras (5.8 [2.9, 12.2] vs. 4.0 [1.9, 9.7] days, *P* = 0.06) in the TF2 era. Survival to CRRT discontinuation did not differ between eras (68% vs. 69%). The lack of association was maintained in subset analyses of patients receiving CRRT in the first 14 days of ICU admission (Table 2) or for their first RAI+ admission (Supplementary Table S1).

### CRRT Discontinuation to ICU Discharge and ICU LOS (CRRT Survivors Only)

CRRT survivor ICU LOS after CRRT discontinuation and total ICU LOS were 6 and 11 days shorter, respectively, in the TF2 era (both *P* < 0.02). CRRT patient survival rates to ICU discharge were higher in the post-TF2 era (*P* = 0.01) (Table 2). Except for ICU LOS, associations were maintained in subset analyses of patients receiving CRRT in the first 14 days of ICU admission (Table 2) or in their first RAI+ admission (Supplementary Table S1).

### Other Analyses

No difference in RAI+ rates was seen for patients on CRRT in the pre-TF2 (calculated manually *post hoc*) versus TF2 era (69% vs. 68%, *P* = 0.88). Analysis of RAI+ patients in both eras showed associations between TF2 era and outcomes were maintained, except ICU survival rates (*P* = 0.06) and ICU LOS (*P* = 0.07) for CRRT survivors (Supplementary Table S3).

**Figure 3:**
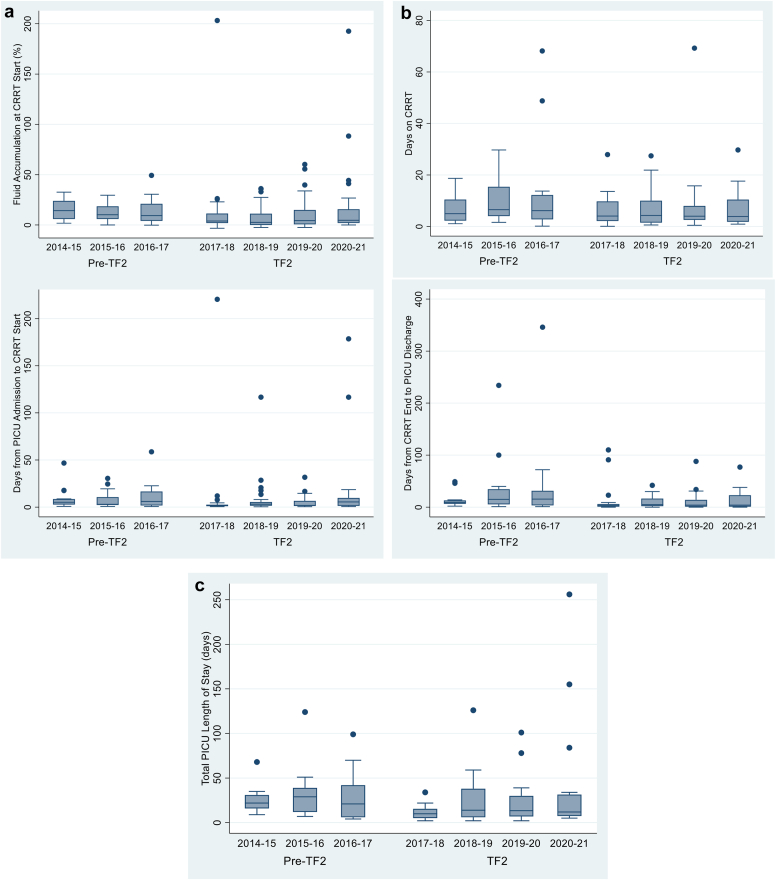
Annual rates or median values for primary and secondary outcomes. All columns represent median values, error bars represent 25th and 75th quartiles and dots represent individual outlier patients. TAKING FOCUS 2 was implemented July 1, 2017, and data are presented through December 31, 2021. (a) Annual Pre-CRRT Initiation Comparisons Between the Pre-TF2 and TF2 Eras. (b) Annual CRRT Treatment Course Parameters (CRRT Survivors Only). (c) Annual PICU length of stay (days). CRRT, continuous renal replacement therapy; PICU, pediatric intensive care unit; TF2, TAKING FOCUS 2.

Year-to-year assessment revealed the decrease in time to CRRT initiation, fluid accumulation, ICU LOS, and mortality in the year of TF2 implementation was sustained in all subsequent years (Figure 3 and Supplementary Table S4). We estimate that TF2 implementation saved $294.49 in CRRT costs (reduced by 1.8 days) and $12,198 in ICU costs (including cost addition of the NGAL test) per surviving CRRT patient (reduced by 5.4 days after CRRT discontinuation).

Eighteen of the 142 patients (12.7%) classified as uNGAL+ were anuric, and thus had an imputed uNGAL > 500 ng/ml. Only 2.8% of TF2 era uNGAL- versus 33.8% of uNGAL+ patients received CRRT by day 7 (Supplementary Table S5) and all 18 anuric patients (classified as uNGAL+) received CRRT by day 7. Excluding anuric patients, 23.4% RAI+/NGAL+ and 42.7% of RAI+/uNGAL >500 ng/ml patients received CRRT, respectively.

Integration of NGAL+ to RAI+ patients improved the positive predictive value for day 2 to 4 severe AKI to RAI+ alone (Supplementary Table S6A). We did not observe a difference in RAI+uNGAL predictive performance stratified by biological sex (Supplementary Table S6B).

## Discussion

This prospective single center study translates and builds upon our previous work integrating risk stratification with a well-studied kidney tubular biomarker, which was limited in scope to improving severe AKI prediction early during a child’s ICU course. The current work applies the information at the bedside as integrated CDS to affect patient outcomes. Provision of RAI and uNGAL data guided by the TF2 pathway was associated with a durable 4-year reduction in time to CRRT initiation, fluid accumulation prior to CRRT initiation, ICU LOS after CRRT discontinuation, and total ICU LOS. We observed increased ICU survival rates for patients on CRRT in the TF2 era. Importantly, only 3% of patients received CRRT other than for NGAL+ status or anuria, whereas one-third RAI+/NGAL+/anuric patients received CRRT. Therefore, it appears clinical teams are not initiating CRRT based on RAI and NGAL alone but are putting the data into clinical context for fluid management/RRT decision making. Finally, we provide a conservative cost savings estimate of $12,507.49 per patient receiving CRRT after TF2 implementation.

Given the study design, the observed outcome improvements can be viewed as only associations in the absence of a randomized controlled trial. However, the preponderance of published observational associations with fluid accumulation at CRRT initiation and poor outcomes^17^ has led to lack of equipoise in the pediatric critical care nephrology community that presents a barrier to randomized study enrollment. Such a dilemma is not restricted to the AKI population; however, numerous studies show improvement in patient outcomes after implementation of a CDS algorithm or standardized practice change. Al-Jaghbeer *et al.*^32^ observed a 34% risk reduction for dialysis and a 24% mortality risk reduction after AKI alert CDS program implementation at their single center. Selby *et al.*^33^ realized decreased hospital LOS and improvements in AKI recognition, medication optimization, and fluid balance assessment with a multifaceted AKI alert, management bundle, and education program implemented sequentially across 5 hospital systems in the United Kingdom. In the absence of a randomized controlled trial, leveraging the RAI-uNGAL-furosemide stress test model at other centers may allow additional data to bolster model performance and improve AKI outcomes. We suggest a step-wedge design similar to the approach taken by Selby *et al.*,^33^ or a cluster randomized trial would be feasible and ethical in the current clinical consensus environment.

Our study was a prospective evaluation over a long period of time, comparing more than 3 years before and after TF2 implementation. The information provided followed optimal informatics CDS guidelines (utilizing the “The Five Rights of CDS”),^34^ by sharing and integrating the following: (i) right information, with (ii) the right caregivers, (iii) at the right time, via the (iv) right media channel, and (v) right format. The TF2 CDS program was shown to be sustainable based on the automated structure and associated cost savings realized. Finally, we leveraged TF2 to investigate other biomarkers (e.g., plasma direct renin concentrations and urine olfactomedin-4)^35^, ^36^ to assess the potential for response to angiotensin II and furosemide, respectively. It is important to note that although the TF2 algorithm provides information (RAI status, NGAL results and fluid accumulation status) to aid in clinical decisions making, it neither mandates fluid accumulation thresholds below or above which CRRT cannot or must be initiated, respectively. Indeed, even though the rate of CRRT initiation below the 15% fluid accumulation threshold decreased after TF2 implementation, 20.6% had CRRT initiated at >15% volume accumulation, and 18.7% had CRRT initiated at >20% volume accumulation.

We acknowledge limitations to our study. First, although performance of RAI and RAI-uNGAL have been validated in multiple studies, the clinical improvements we observed must be validated in multicenter cohorts. Second, we did not have strict 100% adherence to CRRT initiation at <15% fluid accumulation; however, we did observe a sustained 50% reduction in incidence of patients initiating CRRT at >15% accumulation. In complex clinical situations such as AKI with multiorgan failure requiring CRRT, although CDS such as TF2 provides guidance to CRRT initiation, there may be valid reasons that CRRT was not initiated at <15% ICU fluid accumulation. Third, we used the 15% fluid accumulation threshold to consider CRRT initiation with a goal of preventing 20% fluid accumulation given the ppCRRT data demonstrating 8-fold increased odds of mortality at >20%. We examined the rates of CRRT initiation at >5, >10, >15, and >20% pediatric ICU fluid accumulation and found that rates decreased from the pre-TF2 to TF2 eras with the exception of the 20% threshold (Supplementary Table S7). It is certainly possible that other fluid accumulation thresholds could yield even better outcomes. Since we did not train TF2 to provide clinical decisions support at other thresholds, it would be completely speculative to promote or disregard other thresholds. Indeed, a systematic review of the pediatric literature showed that CRRT patient survival was favored by less fluid accumulation, no matter the fluid accumulation threshold.^17^ Fourth, TF2 development, deployment, calibration, and validation required 2 years and approximately $500,000 of internal and external funding to achieve reliability. Widespread dissemination will require more cost-efficient and time-efficient solutions, even with the potential health care cost savings and better patient-centered outcomes we demonstrated. Fifth, we acknowledge the limitation of not being able to eliminate a potential time effect from changes in case-mix, ICU practice or general improvement in standard of care. We propose that this is not the case, because improvements occurred almost immediately after TF2 implementation and persisted for 4 years. Sixth, another limitation is the lack of a standing codified protocol for CRRT liberation in our program, which obviously impacts outcome measures such as CRRT duration and days from CRRT termination to ICU discharge. In fact, CRRT liberation strategies and criteria represent a well-recognized major knowledge gap in the field^12^; therefore, further validation of the TF2 pathway success will require standardization of CRRT liberation practices. Seventh, our ICU LOS data could be biased by ICU organization and strained capacity. Because we defined ICU LOS by admission and discharge dates (and not by date and time transfer orders were written) we cannot assess the potential impact of delayed discharge on LOS and ICU costs. Finally, our cost analysis was rudimentary, using only average ICU and study costs per patient receiving CRRT. A more granular patient level cost assessment (including medication and supply costs) is planned, but beyond the scope of this manuscript.

Despite these limitations, we suggest that risk stratification to direct biomarker assessment and clinical decisions regarding fluid management guided by the TF2 pathway provides initial evidence for patient-centered and health care resource benefits. The RAI directs uNGAL assessment in only the 10% of patients who could benefit, which improves uNGAL performance and optimizes health care resources by reducing unnecessary AKI-related testing in 90% of admissions. Use of uNGAL thresholds further refines risk assessment, directing CRRT initiation earlier with the potential to decrease CRRT duration, ICU LOS, patient morbidity, and mortality. Initial investments for TF2 deployment should be offset quickly by cost and patient-centered outcome improvements. Future analyses will examine disease-specific RAI thresholds (e.g., sepsis,^37^ post-cardiac surgery^38^); and long-term outcomes for TF2 CRRT survivors, including chronic kidney disease, hypertension, or proteinuria development. Finally, we used the RAI to direct NGAL utilization in TF2, because NGAL is clinically available on our laboratory platform. The performance of RAI directed utilization of other AKI biomarkers (e.g., urine Kidney Injury Molecule-1, insulin-like growth factor-binding protein 7/tissue inhibitor of metalloproteinases-2) should be explored in future studies.

## Disclosure

SLG and RKB consult for and receive research funding from BioPorto Diagnostics, Inc, which markets The NGAL Test. SLG, RKB, and Cincinnati Children’s Hospital Medical Center have licensed the RAI application to RAIDAR Health, Inc, and will receive royalties from sales. LC is a consultant for BioPorto Diagnostics and a Stockholder of RAIDAR Health. Neither BioPorto Diagnostics nor RAIDAR Health had any input into TAKING FOCUS 2, and they did not provide any financial or material support. All other authors have declared no conflicting interest.

## Supplementary Material

Supplementary File (PDF)

**Figure S1.** Pre-TF2 and TF2 clinical decision pathways.

**Table S1.** All RAI+ Patients who received CRRT initial occurrence only.

**Table S2.** Demographics of CRRT survivors versus NonSurvivors.

**Table S3.** Outcomes for RAI+ patients who received CRRT in each era.

**Table S4.** Year over year trends in CRRT related outcomes.

**Table S5.** RAI+/uNGAL classification for TF2 patients who received CRRT.

**Table S6.** (A) RAI+/NGAL Performance to Predict Day 2-4 Severe AKI (sAKI). (B) RAI+/NGAL Performance Stratified by Biological Sex to Predict Day 2-4 Severe AKI (sAKI).

**Table S7.** Comparison of different CRRT initiation fluid accumulation thresholds between the pre-TF2 and TF2 eras.

**STROBE Checklist.**
