# Supplementary material for: Real-Time Acute Kidney Injury Risk Stratification–Biomarker Directed Fluid Management Improves Outcomes in Critically Ill Children and Young Adults
Source: Kidney Int Rep. 2023 Sep 22;8(12):2690–700. doi: 10.1016/j.ekir.2023.09.019 (PMC10719644; doi:10.1016/j.ekir.2023.09.019)
Supplement: Supplementary File (PDF) [file mmc1.pdf]

STROBE Statement—Checklist of items that should be included in reports of ***cohort studies***

	Item No	Recommendation	Page No
**Title and abstract**	1	(*a*) Indicate the study's design with a commonly used term in the title or the abstract<br><br>(*b*) Provide in the abstract an informative and balanced summary of what was done and what was found	(a) 1<br><br>(b) 3
**Introduction**			
Background/rationale	2	Explain the scientific background and rationale for the investigation being reported	4-5
Objectives	3	State specific objectives, including any prespecified hypotheses	5
**Methods**			
Study design	4	Present key elements of study design early in the paper	5-6
Setting	5	Describe the setting, locations, and relevant dates, including periods of recruitment, exposure, follow-up, and data collection	5
Participants	6	(*a*) Give the eligibility criteria, and the sources and methods of selection of participants. Describe methods of follow-up<br><br>(*b*) For matched studies, give matching criteria and number of exposed and unexposed	6
Variables	7	Clearly define all outcomes, exposures, predictors, potential confounders, and effect modifiers. Give diagnostic criteria, if applicable	7
Data sources/ measurement	8*	For each variable of interest, give sources of data and details of methods of assessment (measurement). Describe comparability of assessment methods if there is more than one group	7
Bias	9	Describe any efforts to address potential sources of bias	7-8
Study size	10	Explain how the study size was arrived at	7
Quantitative variables	11	Explain how quantitative variables were handled in the analyses. If applicable, describe which groupings were chosen and why	7-9
Statistical methods	12	(*a*) Describe all statistical methods, including those used to control for confounding<br><br>(*b*) Describe any methods used to examine subgroups and interactions<br><br>(*c*) Explain how missing data were addressed<br><br>(*d*) If applicable, explain how loss to follow-up was addressed<br><br>(*e*) Describe any sensitivity analyses	7-9
**Results**			

Participants	13*	(a) Report numbers of individuals at each stage of study—eg numbers potentially eligible, examined for eligibility, confirmed eligible, included in the study, completing follow-up, and analysed<br>(b) Give reasons for non-participation at each stage<br>(c) Consider use of a flow diagram	9
Descriptive data	14*	(a) Give characteristics of study participants (eg demographic, clinical, social) and information on exposures and potential confounders<br>(b) Indicate number of participants with missing data for each variable of interest<br>(c) Summarise follow-up time (eg, average and total amount)	9-10
Outcome data	15*	Report numbers of outcome events or summary measures over time	9-11

Main results	16	(*a*) Give unadjusted estimates and, if applicable, confounder-adjusted estimates and their precision (eg, 95% confidence interval). Make clear which confounders were adjusted for and why they were included<br><br>(*b*) Report category boundaries when continuous variables were categorized<br><br>(*c*) If relevant, consider translating estimates of relative risk into absolute risk for a meaningful time period	9-10
Other analyses	17	Report other analyses done—eg analyses of subgroups and interactions, and sensitivity analyses	11
**Discussion**			
Key results	18	Summarise key results with reference to study objectives	11
Limitations	19	Discuss limitations of the study, taking into account sources of potential bias or imprecision. Discuss both direction and magnitude of any potential bias	12
Interpretation	20	Give a cautious overall interpretation of results considering objectives, limitations, multiplicity of analyses, results from similar studies, and other relevant evidence	12-13
Generalisability	21	Discuss the generalisability (external validity) of the study results	14
**Other information**			
Funding	22	Give the source of funding and the role of the funders for the present study and, if applicable, for the original study on which the present article is based	15

*Give information separately for exposed and unexposed groups.

Note: An Explanation and Elaboration article discusses each checklist item and gives methodological background and published examples of transparent reporting. The STROBE checklist is best used in conjunction with this article (freely available on the Web sites of PLoS Medicine at http://www.plosmedicine.org/, Annals of Internal Medicine at http://www.annals.org/, and Epidemiology at http://www.epidem.com/). Information on the STROBE Initiative is available at http://www.strobe-statement.org.

Supplementary Figure S1 – Pre-TF2 and TF2 Clinical Decision Pathways

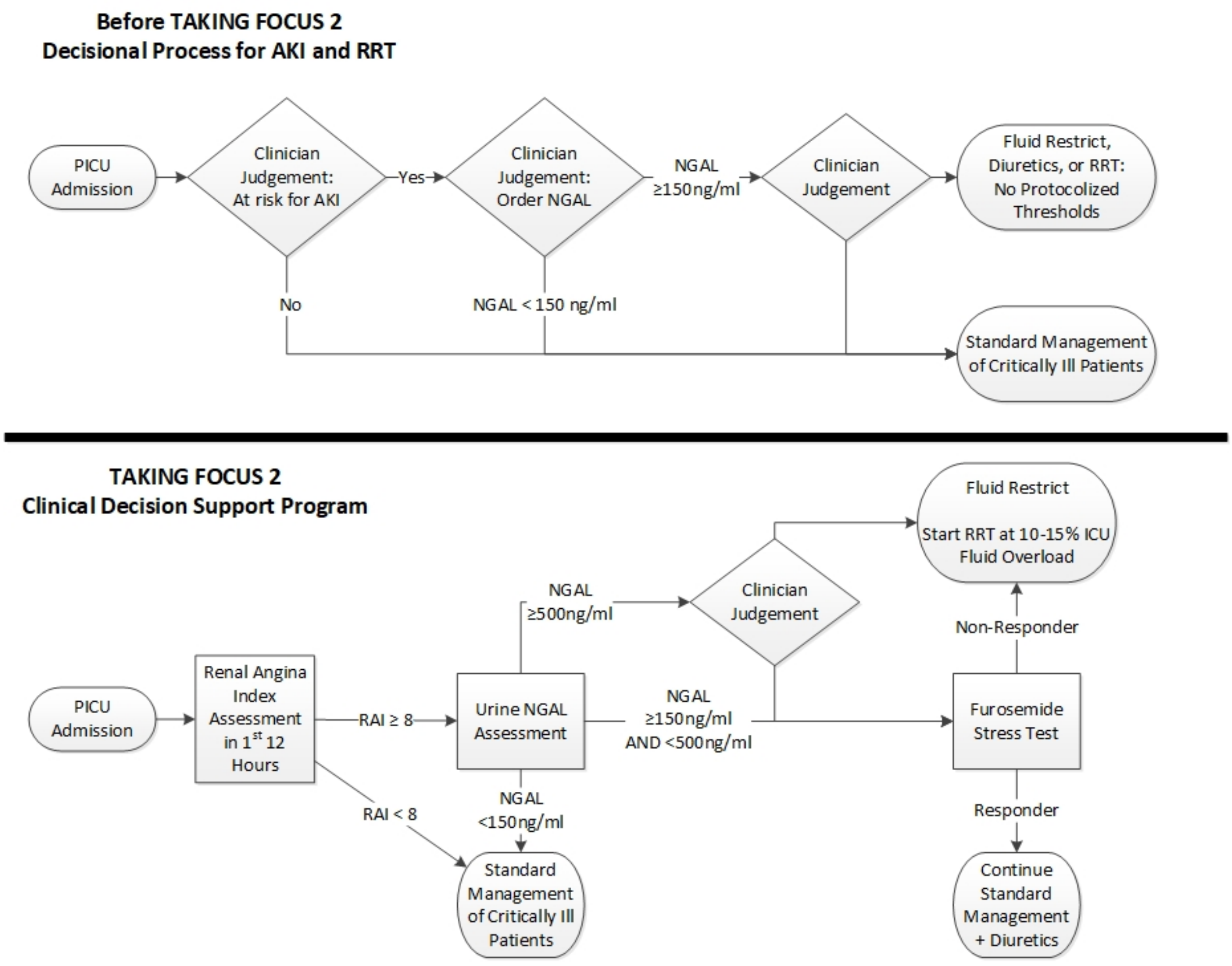

Supplementary Table S1 – All RAI+ Patients who Received CRRT Initial Occurrence Only (n=159)

All RAI+ Patients who Received CRRT Initial Occurrence Only (n=159)				
**Variable**		**Pre-TF2 (n=68)**	**TF2 (n=91)**	**p-value**
**Pre-CRRT Patient Demographics and Fluid Status**				
Time from PICU admission to CRRT initiation (days)	Median [IQR]	4.2 [2.5, 11.8]	2 .6 [1.5, 6.6]	0.002
Fluid Accumulation from PICU admission to CRRT Initiation (%)	Median [IQR]	12.8 [5.9, 20.4]	6.2 [1.4, 14.9]	0.01
PICU Fluid Accumulation >15% at CRRT Initiation	Yes	22 (39.3%)	18 (18.9%)	0.008
**Patient Outcome Measures**		**Pre-TF2 (n=68)**	**TF2 (n=91)**	
Survival to CRRT Discontinuation	Yes	36 (64.3%)	68 (71.6%)	0.37
Survival to PICU Discharge	Yes	26 (46.4%)	65 (68.4%)	0.01
		**Pre-TF2 (n=36)**	**TF2 (n=68)**	
CRRT Duration among CRRT Survivors (days)	Median [IQR]	5.8 [3.2, 12.2]	4.1 [2,2, 9.7]	0.06
		**Pre-TF2 (n=26)**	**TF2 (n=65)**	
PICU LOS after CRRT D/C among PICU Survivors (days)	Median [IQR]	7.9 [2.5, 11]	2.5 [0.6, 6.5]	0.01
Total PICU LOS among PICU Survivors (days)	Median [IQR]	17 [9, 31]	11 [6, 20]	0.08

a. PRISM – Pediatric Risk of Mortality Score
b. LOS – Length of Stay

Supplemental Table S2 – Demographics of CRRT Survivors *vs.* Non-Survivors

	**All patients**		
	**Survivors (n=122)**	**Non-Survivors (n=56)**	**p-value**
Patient age (years)	10.3 [2.2, 15.8]	8.8 [2.7, 16.7]	0.86
Patient weight (kg)	29.5 [12.6, 54.3]	29.1 [13.8, 53.5]	0.95
PRISM III Score	10 [6, 16]	13 [9, 17]	0.07
Time from PICU admission to CRRT initiation (days)	2.6 [1.5, 6.7]	4.1 [1.6, 10.1]	0.18
Fluid Accumulation from PICU admission to CRRT Initiation (%)	6.7 [1.9, 15.8]	7.1 [3.1, 16.4]	0.45
	**TF2 Cohort Only**		
	**Survivors (n=74)**	**Non-Survivors (n=33)**	**p-value**
Patient age (years)	10.1 [2.3, 17]	10.8 [2.1, 16.8]	0.84
Patient weight (kg)	30.9 [13.6, 56.6]	13.2 [13.2, 61.1]	0.94
PRISM III Score	9.5 [5, 17]	13 [8, 17]	0.26
Time from PICU admission to CRRT initiation (days)	1.7 [1.1, 4.7]	3.6 [1.5, 9.7]	0.05
Fluid Accumulation from PICU admission to CRRT Initiation (%)	3.5 [0.4, 12.2]	6.3 [2.2, 12.1]	0.31

a. All values median [IQR]
b. PRISM – Pediatric Risk of Mortality Score

Supplementary Table S3 – Outcomes for RAI+ patients who Received CRRT in Each Era

All RAI+ Patients who Received CRRT (n=105)				
**Variable**		**Pre-TF2 (n=43)**	**TF2 (n=62)**	**p-value**
**Pre-CRRT Patient Demographics and Fluid Status**				
Time from PICU admission to CRRT initiation (days)	Median [IQR]	4 [3, 11]	2 [1, 4]	0.003
Fluid Accumulation from PICU admission to CRRT Initiation (%)	Median [IQR]	12.1 [4.0, 18.6]	4.1 [0.6, 12.2]	0.008
PICU Fluid Accumulation >15% at CRRT Initiation	Yes	15 (34.8%)	8 (12.9%)	0.007
**Patient Outcome Measures**		**Pre-TF2 (n=43)**	**TF2 (n=62)**	
Survival to CRRT D/C	Yes	29 (67.4%)	41 (66.1%)	0.89
Survival to PICU D/C	Yes	20 (46.5%)	40 (64.5%)	0.07
		**Pre-TF2 (n=29)**	**TF2 (n=41)**	
CRRT Duration among CRRT Survivors (days)	Median [IQR]	5.5 [2.7, 10.5]	4.1 [2.4, 9.1]	0.01
		**Pre-TF2 (n=20)**	**TF2 (n=40)**	
PICU LOS after CRRT D/C among PICU Survivors (days)	Median [IQR]	7.9 [2.6, 13.2]	2.4 [0.7, 6.2]	0.01
Total PICU Length of stay among PICU Survivors (days)	Median [IQR]	20 [9.5, 35.5]	11 [6, 19]	0.06

Supplementary Table S4 – Year over Year Trends in CRRT Related Outcomes[a,b]

Variable	Category	2014	2015	2016	2017[b]	2018	2019	2020
**Time from PICU admission to CRRT initiation (days)**	**n**	**20**	**27**	**25**	**26**	**30**	**28**	**23**
	Median [IQR]	5.5 [3,9]	3 [3,11]	7 [2,17]	2 [2,3]	3.5 [2,6]	2 [2,7]	6 [2,10]
**Fluid Accumulation from PICU admission to CRRT Initiation (%)**	**n**	**20**	**27**	**25**	**26**	**30**	**28**	**23**
	Median [IQR]	14.4 [6, 24]	10.3 [6,18.6]	6.9 [4.3,19.6]	4 [2, 11.5]	2.7 [0,11.3]	4.4 [0.6, 15]	4.8 [2.1,15.7]
**PICU Fluid Accumulation >15% at CRRT Initiation**	Yes	8 (40%)	10 (37%)	8 (32%)	5 (19%)	4 (13%)	7 (25%)	6 (26%)
**Survival to CRRT D/C**	Yes	12 (60%)	20 (74%)	16 (64%)	18 (69%)	20 (67%)	19 (68%)	17 (74%)
**Survival to PICU D/C**	Yes	9 (45%)	13 (48%)	11 (44%)	16 (62%)	20 (67%)	18 (64%)	16 (70%)
**CRRT Duration among CRRT Survivors (days)**	**n**	**12**	**20**	**16**	**18**	**20**	**19**	**17**
	Median [IQR]	5 [2.3,10.5]	6.5 [4.1,15.4]	6 [2.8,12.2]	4 [2.2,9.8]	4 [1.5,10]	4 [2.6, 8]	4 [1.9,10.5]
**PICU LOS after CRRT D/C among PICU Survivors (days)**	**n**	**9**	**13**	**11**	**16**	**20**	**18**	**16**
	Median [IQR]	9 [9,12]	11 [5,23]	7 [2,24]	2.5 [1,4.5]	5 [2,16.5]	3.5 [1,11]	3 [1,17]
**Total PICU Length of stay among PICU Survivors (days)**	**n**	**9**	**13**	**11**	**16**	**20**	**18**	**16**
	Median [IQR]	22 [16,35]	29 [12,39]	21 [6,42]	10 [5,15.5]	14 [6,38]	13.5 [7,30]	12 [7.5,31.5]

a. Years represent July of the year represented through June of the following year.
b. TAKING FOCUS 2 was initiated July 1, 2017.

Supplementary Table S5 – RAI+/uNGAL Classification for TF2 Patients Who Received CRRT

Classification	CRRT (Yes)	CRRT (No)	Total
RAI+/uNGAL-	3 (2.8%)	103 (97.2%)	**106**
RAI+/uNGAL+	48 (33.8%)	94 (66.2%)	**142**
Total	**51**	**197**	

Supplementary Table S6a – RAI+/NGAL Performance to Predict Day 2-4 Severe AKI (sAKI)

Tested result	n	PPV (95%CI)	Sensitivity (95%CI)	(+) Likelihood Ratio (95%CI)	(-) Likelihood Ratio (95% CI)
RAI+	304	0.50 (0.44- 0.56)			
NGAL results*	248				
NGAL+	142	0.75 (0.67-0.82)	0.78 (0.70-0.84)	2.44 (1.83 - 3.25)	
NGAL-	106				0.33 (0.24 - 0.46)

***uNGAL+ is >150 ng/mL, uNGAL- is ≤150 ng/mL**

Supplementary Table S6b – RAI+/NGAL Performance Stratified by Biological Sex to Predict Day 2-4 Severe AKI (sAKI)

Sex	NGAL results* (N=248)	PPV D2-4 AKI	PPV 95% CI	NPV D2-4 No AKI	NPV 95% CI
Male	NGAL+ (N=75)	59/75=0.79	(0.68,0.87)		
	NGAL- (N=65)			46/65=0.71	(0.58,0.81)
Female	NGAL+ (N=67)	48/67=0.72	(0.59,0.82)		
	NGAL- (N=41)			29/41=0.71	(0.53,0.84)
	D2-4 AKI	NGAL+ sensitivity	95% CI	NGAL- specificity	95% CI
Male	AKI+	59/78=0.76	(0.65, 0.85)		
	AKI-			46/62= 0.74	(0.62,0.84)
Female	AKI+	48/60=0.80			
	AKI-		(0.68, 0.89)	29/48=0.60	(0.45,0.74)

***uNGAL+ is >150 ng/mL, uNGAL- is ≤150 ng/mL**

Supplementary Table S7 – Comparison of Different CRRT Initiation Fluid Accumulation thresholds between the pre-TF2 and TF2 eras

Fluid Accumulation at CRRT Initiation Threshold		Pre-TF2 n=71	TF2 n=107	p-value
**>5%**	**Yes**	53 (74.6%)	49 (45.8%)	0.0002
**>10%**		38 (53.5%)	34 (31.8%)	<0.005
**>15%**		26 (36.6%)	22 (20.6%)	<0.03
**>*20*%**		18 (25.4%)	20 (18.7%)	0.36
